# Resampling and harmonization for mitigation of heterogeneity in image parameters of baseline scans

**DOI:** 10.1038/s41598-022-26083-4

**Published:** 2022-12-13

**Authors:** Apurva Singh, Hannah Horng, Rhea Chitalia, Leonid Roshkovan, Sharyn I. Katz, Peter Noël, Russell T. Shinohara, Despina Kontos

**Affiliations:** 1 Department of Radiology, University of Pennsylvania, Philadelphia, PA 19104 USA; 2 Department of Bioengineering, University of Pennsylvania, Philadelphia, PA 19104 USA; 3 Department of Biostatistics, Epidemiology, and Informatics, University of Pennsylvania, Philadelphia, PA 19104 USA; 4 Department of Radiology, Center for Biomedical Image Computing and Analytics (CBICA), University of Pennsylvania, Rm D702 Richards Bldg., 3700 Hamilton Walk, Philadelphia, PA 19104 USA

**Keywords:** Computational biology and bioinformatics, Biomarkers

## Abstract

Our study investigates the effects of heterogeneity in image parameters on the reproducibility of prognostic performance of models built using radiomic biomarkers. We compare the prognostic performance of models derived from the heterogeneity-mitigated features with that of models obtained from raw features, to assess whether reproducibility of prognostic scores improves upon application of our methods. We used two datasets: The Breast I-SPY1 dataset—Baseline DCE-MRI scans of 156 women with locally advanced breast cancer, treated with neoadjuvant chemotherapy, publicly available via The Cancer Imaging Archive (TCIA); The NSCLC IO dataset—Baseline CT scans of 107 patients with stage 4 non-small cell lung cancer (NSCLC), treated with pembrolizumab immunotherapy at our institution. Radiomic features (n = 102) are extracted from the tumor ROIs. We use a variety of resampling and harmonization scenarios to mitigate the heterogeneity in image parameters. The patients were divided into groups based on batch variables. For each group, the radiomic phenotypes are combined with the clinical covariates into a prognostic model. The performance of the groups is assessed using the c-statistic, derived from a Cox proportional hazards model fitted on all patients within a group. The heterogeneity-mitigation scenario (radiomic features, derived from images that have been resampled to minimum voxel spacing, are harmonized using the image acquisition parameters as batch variables) gave models with highest prognostic scores (for e.g., IO dataset; batch variable: high kernel resolution—c-score: 0.66). The prognostic performance of patient groups is not comparable in case of models built using non-heterogeneity mitigated features (for e.g., I-SPY1 dataset; batch variable: small pixel spacing—c-score: 0.54, large pixel spacing—c-score: 0.65). The prognostic performance of patient groups is closer in case of heterogeneity-mitigated scenarios (for e.g., scenario—harmonize by voxel spacing parameters: IO dataset; thin slice—c-score: 0.62, thick slice—c-score: 0.60). Our results indicate that accounting for heterogeneity in image parameters is important to obtain more reproducible prognostic scores, irrespective of image site or modality. For non-heterogeneity mitigated models, the prognostic scores are not comparable across patient groups divided based on batch variables. This study can be a step in the direction of constructing reproducible radiomic biomarkers, thus increasing their application in clinical decision making.

## Introduction

A well-rounded profiling of the properties of the tumor regions is the primary goal of precision cancer medicine, that plays a crucial role in deciding the suitable course of therapy^1^. Tumor characterization using genomic and molecular profiling is not performed in a routine manner due to the expensive and time-consuming nature of the procedures^2^. The assessment of the properties of the tumor regions using a traditional tissue biopsy is also limited in accuracy, owing to the heterogeneous nature of the tumor regions. A small sample of the tumor is not fully reflective of the properties of the entire tumor region, and cannot characterize the change in the tumor properties over time. Further, repeated tissue sampling at various treatment time points may expose the patients to potential procedure-related complications, due to its invasive nature^3^.

Medical imaging, historically used as a diagnostic tool, is increasingly becoming popular in the field of personalized medicine, as an alternative to genomic and proteomic technologies, as it provides a macroscopic and non-invasive view of the tissues of interest^4,5^. Radiomics focuses on extracting quantitative data from medical images, that help decode biological information by quantifying their phenotypic characteristics in a high-throughput manner, allowing these features to function as biomarkers^6,7^. With improvement in feature extraction techniques, large numbers of quantitative radiomic features can now be analyzed. This allows better characterization of the properties of the heterogeneous tumor regions^8^. For instance, Huang et al. developed a radiomic signature from preoperative CT images to predict lymph node metastasis in patients with colorectal cancer^9^. Velazquez et al. developed a prognostic model using radiomic signatures from pre and post-radiotherapy FDG-PET-CT scans of patients with advanced NSCLC and combining them with clinical factors, and used it to identify patients at risk of residual disease^10^.

While the applications of radiomics are promising, the implementation of radiomics into clinical routine remains challenging. Biomarkers are defined as “the objective indications of medical state observed from outside the patient- that can be measured reproducibly”^11^. This implies that biomarkers must remain comparable, even after subtle changes in the measurement process. Radiomic features are essentially mathematical equations applied to numerical arrays of intensity values that form the medical image^12^. Thus, changes in the values in the array (due to differences in scan acquisition and reconstruction parameters), lead to potentially significant quantitative changes in the features. This makes it difficult to obtain stable, replicable results from the prognostic radiomic biomarkers^13^. It is also difficult to establish if the quantitative changes in the radiomic biomarkers are due to actual physiological variations or the heterogeneity in image parameters. Thus, this inability of the radiomic biomarkers to be quantified in a reproducible manner has made it difficult for them to gain widespread acceptance in routine clinical decision-making^14^.

Efforts are now being made in the radiomics community to better understand the sources of variation in image parameters, to improve the reproducibility and transparency of the observations obtained from these studies. Some studies have reported on the sensitivity of radiomic features to test–retest variability, in which two scans of a patient (or a phantom) are taken after a time interval using the same scanning parameters. Timmermen et al. performed a test–retest analysis on CT scans of 40 patients with rectal cancer in a clinical setting (pre-treatment scans, with a median interval of 8 days between them). The correlation between radiomic features was assessed using the concordance correlation coefficient (CCC). These results were compared to the test–retest results on CT scans of 27 patients with lung cancer, with a 15-miute interval (“coffee-break” test–retest setting^15^) between the scans. In total, 82.3% of the features have a higher CCC for the test–retest analysis of the dataset of patients with lung cancer than for patients with rectal cancer. The results indicate that radiomic feature robustness varies according to tumor site, and varies between the traditional “coffee-break” and clinical test–retest settings^16^.

Radiomic studies have now begun to explore the sensitivity of radiomic features to the variation in image acquisition and reconstruction protocols, inter-observer segmentation variability, and other technical factors. For instance, Zhovannik et al. aimed to characterize the variation in radiomic feature distribution due to differences in scanner signal to noise ratio (SNR). They used a phantom with 17 regions of interest (ROIs) and the scans were acquired with nine exposure settings. Results showed that roughly two-thirds of the radiomic features depend on the exposure settings of the scanner^17^. Midya et al. assessed the sensitivity of radiomic features extracted from phantom scans to the variation in tube current and noise index levels. They used the CCC metric to assess the agreement of features^18^. Mackin et al. assessed the variation in radiomics features obtained from 17 scans of a radiomics phantom, acquired using scanners from different manufacturers, using varying imaging protocols. The variation in features was captured using a “feature noise” metric^19^. These studies emphasized the importance of minimizing inter-scanner differences for improving radiomic feature reproducibility.

Recent radiomic studies have also looked at Combining Batches (ComBat) harmonization methods to address the effect of image parameter heterogeneity on the reproducibility of radiomic features. ComBat, originally introduced for gene expression analysis, is a method that was introduced for removing the effects of machinery and protocols used to extract gene expression data, to make the data acquired from various centers comparable^20^. Ibrahim et al. investigated the reproducibility of radiomic features across the scans of a multi-layer phantom, acquired using different scanners, by assessing the number (%) of reproducible radiomic features before vs. after ComBat harmonization. The radiomic features extracted from thirteen scans of the ten-layer phantom (each layer had sixteen ROIs) were compared in a pair-wise manner. The number (%) of reproducible radiomic features (varying across the pairwise scenarios, determined using the CCC metric) was higher for harmonized features (range 15.4–87.9%) when compared to the non-harmonized features (range 8.8 to 85.7%)^21^.

The studies discussed above have focused on the reproducibility of the radiomic features in a limited setting (test–retest scenarios, phantom studies and so on). The variation in radiomic features in a test–retest experimental scenario (difference between the scans is in the order of minutes) is not reflective of a test–retest scenario in a clinical setting (difference between the scans is in the order of days), as significant physiological changes in the tumor regions can over time. Similarly, the assessment of the effect of image parameter heterogeneity on the reproducibility of radiomic features extracted from phantom scans is not comparable to the assessment performed on the features extracted from tumor regions. This is because the features extracted from human tissue are expected to encapsulate a wider range of variation, as they are also influenced by biological factors. Further, while the studies have explored the sensitivity of individual radiomic features to image parameter variation, little attention has been given to assessing how the image parameter heterogeneity affects the reproducibility of radiomic biomarkers, and how various heterogeneity-mitigation techniques can be used to improve the robustness of the radiomic signatures.

Our study aims to investigate the effects of individual image parameters and how their heterogeneity affects the reproducibility of prognostic performance of models built using radiomic biomarkers. We have used a variety of resampling and harmonization techniques to mitigate the heterogeneity in the radiomic features. We will compare the prognostic performance of the models derived from the heterogeneity-mitigated features with the performance of the models obtained from the raw, non-heterogeneity mitigated features, and assess whether the reproducibility of the prognostic scores improves upon the application of our methods. We hypothesize that the radiomic biomarkers derived from images with more homogenous imaging parameters will produce models whose prognostic performance is more consistent across the individual parameter categories. Our study includes two databases. The first dataset consists of baseline DCE-MRI scans of 156 women with locally advanced breast cancer, publicly available via The Cancer Imaging Archive (TCIA). The women underwent neoadjuvant chemotherapy with an anthracycline-cyclophosphamide regimen alone or followed by taxane. The second dataset consists of baseline CT scans of 107 patients with stage 4 NSCLC, treated at our institution with first-line pembrolizumab monotherapy or combination therapy. We have included datasets from different organ sites and different image modalities, to see if our hypothesis holds across different sites and modalities.

## Materials and methods

### Breast I-SPY1 dataset

#### Study sample and data

The ACRIN 6657/I-SPY1 TRIAL^22^ enrolled n = 237 women from May 2002 to March 2006. From this cohort, n = 230 women met the eligibility criteria of being diagnosed with locally advanced breast cancer with primary tumors of stage T3 measuring at least 3 cm in diameter. The pre-operative DCE-MRI images of 222 women were publicly available via The Cancer Imaging Archive (TCIA)^23^. From this TCIA set, 15 women were excluded for our present study, due to incomplete DCE acquisition scans. A subsequent 51 women were also excluded due to either incomplete histopathologic data or recurrence-free survival (RFS) outcome, or missing pre-treatment DCE-MRI scans. This resulted in the inclusion of n = 156 women for this study, with baseline DCE-MRI scans. All women underwent longitudinal DCE-MRI imaging on a 1.5 T field-strength system. Women underwent neoadjuvant chemotherapy with an anthracycline-cyclophosphamide regimen alone or followed by taxane. The demographic information of the patients is included in Supplementary Table S1.

### NSCLC IO dataset

#### Study sample and data

This single-center retrospective, observational study was conducted at the Hospital of the University of Pennsylvania between November 2016 and December 2020. The study was approved by the University of Pennsylvania’s Institutional Review Board (IRB) committee under a waiver of informed consent. All methods in this study were in accordance with the Declaration of Helsinki. Patients (n = 107) with stage 4 Non-Small Cell Lung Cancer (NSCLC) treated with first-line pembrolizumab based therapy at our institution were identified. The demographic information of the patients is included in Supplementary Table S3. Preliminary analyses conducted by our group on this dataset can be found here^24^.

#### Radiomic feature extraction

The 3D tumor volumes were manually segmented by board-certified, fellowship-trained radiologists using the semi-automated ITK-SNAP software (version 3.6.0)^25^. We have used the Cancer Phenomics Toolkit (CaPTk)^26^, a highly-standardized, user-friendly, open-source software developed at our institution, that conforms to the Imaging Biomarker Standardization Initiative (IBSI) radiomics standardization protocols^27^, for extraction of radiomic features (n = 102) from the tumor regions of interest (ROIs). The radiomic features represent the following eight type of descriptors: (1) Intensity features or first-order statistics (capturing the voxel grey-level intensities within a neighborhood). (2) Histogram-based features (computed using an intensity histogram by discretization of the original intensity distribution. (3) Volumetric features (computed by utilizing the voxel intensities in the ROI and are based on the relationship between discretized intensity and the fraction of the volume containing the least intensity). (4) Morphologic features (describe geometric aspects of a region of interest (ROI), such as area and volume). (5) Gray level run length matrix features (based on quantifying gray level runs as the lengths of consecutive pixels). (6) Neighboring gray tone difference matrix features (rotation-independent features based on gray-level relationships between neighboring voxels and aim to capture the coarseness of the overall texture). (7) Gray level size zone matrix features (grey level size zone matrix (GLSZM) counts the number of groups (or zones) of linked voxels, where voxels are linked if the neighboring voxel has an identical discretized grey level). (8) Local binary pattern features (describe the local texture patterns in an image where the LBP works in a block size of 3 × 3, in which the center pixel is used as a threshold for the neighboring pixel, and the LBP code of a center pixel is generated by encoding the computed threshold value into a decimal value). A list of features belonging to each family and their formulae, can be found in Supplementary Tables S13 and S14 respectively.

#### Radiomic feature harmonization

ComBat is a harmonization method originally developed for genomics that can correct variation in features due to imaging parameters by using empirical Bayes to estimate location and scale parameters to shift data^28^. While ComBat is fast and easy to use, current implementations of ComBat are only able to harmonize by a single batch effect at a time and are therefore unable to adequately harmonize datasets that are heterogeneous in more than one batch effect. The OPNested ComBat approach used in our study enables harmonization by multiple batch effects by implementing sequential harmonization^29–31^. The approach was initialized with the radiomic features as input data and a list of batch variables (Breast I-SPY1: Table 1 and NSCLC IO: Table 2). The outcome variables (death and overall survival (breast ISPY1 dataset) and recurrence event and months of progression-free survival (lung IO dataset) and clinical covariates (age, HR Pos and HER2Most Pos (Breast ISPY1 Dataset, Supplementary Table S2) and age, sex, race, PD-L1 expression, ECOG status, BMI and smoking status (Lung IO Dataset, Supplementary Table S4)) were all protected during harmonization to prevent the removal of biological variables of interest. The harmonized feature set with the lowest number of features with detected differences in distribution across all batch effects using the Anderson–Darling (AD) test was selected as the final output. Features remaining significantly affected by batch effects after ComBat harmonization as detected with the AD test were discarded from further analysis. The percentage of features with significantly different distributions arising from the batch effects was reduced after applying harmonization to the original features (Supplementary Tables S5, S6).

**Table 1:** List of batch effects: breast I-SPY1 dataset.

Batch effect	Category	Number of patients (n = 156)
Clinical site	Site AAB	25 (16.1%)
	Site AAC	3 (1.9%)
	Site AAD	5 (3.2%)
	Site AAE	31 (19.9%)
	Site AAG	10 (6.4%)
	Site AAH	20 (12.8%)
	Site AAI	62 (39.7%)
Manufacturer	GE	106 (67.9%)
	Philips	11 (7.1%)
	Siemens	39 (25%)
Scanner name	Genesis_signa	93 (59.6%)
	Intera	11 (7.1%)
	Magnetom Vision	16 (10.3%)
	Magnetom Vision Plus	3 (1.9%)
	Signa excite	13 (8.3%)
	Sonata	20 (12.8%)
Pixel spacing Dynamic range: [0.7 mm, 1.13 mm]	< 0.78 mm	57 (36.5%)
	≥ 0.78 mm	99 (63.5%)
Slice thickness Dynamic range: [1.5 mm, 3.5 mm]	< 2.1 mm	74 (47.4%)
	≥ 2.1 mm	82 (52.6%)

**Table 2:** List of batch effects: NSCLC IO dataset.

Batch effect	Category	Number of patients (n = 107)
Contrast enhancement	Contrast-enhanced	80 (74.8%)
	Non-contrast-enhanced	27 (25.2%)
Kernel resolution (Manufacturer)	Low Resolution-Soft tissue kernel (≤ B40f (Siemens), B, C, D (Philips), STD (GE))	90 (84.1%)
	High Resolution-Lung Kernel (> B40f (Siemens), A (Philips), LUNG (GE))	17 (15.8%)
Pixel spacing Dynamic range (mm): [0.54, 1.17]	< 0.75 mm	58 (54.2%)
	≥ 0.75 mm	49 (45.8%)
Slice thickness Dynamic range (mm): [0.8, 3.75]	< 1.5 mm	64 (59.8%)
	≥ 1.5 mm	43 (40.2%)

#### Accounting for heterogeneity in imaging parameters

In our study, we use the following scenarios (summarized in Table 3) to mitigate the heterogeneity in image parameters:

1. The variation in image physical dimensions is addressed by harmonizing the radiomic features using the voxel spacing parameters as the batch variables. This is performed under two scenarios, using offsets of 3 mm (1A) or 5 mm (1B) while extracting the features. Here, offset defines the distance between the center voxel and the neighboring voxels.
  We keep a common offset value for feature extraction, since the voxel spacing varies across the images. Thus, a standard offset value (either 3 mm or 5 mm) will ensure the feature extraction is being performed in the same physical dimension.
2. The variation in image acquisition parameters is addressed by harmonizing the radiomic features using contrast enhancement and kernel resolution as the batch variables. This is performed under two scenarios, using offsets of 3 mm (2A) or 5 mm (2B) while extracting the features.
3. The variation in the image physical dimension (voxel spacing) parameters is addressed by performing anisotropic resampling on the images. The images are resampled to the minimum value across each of the voxel spacing parameters [Breast I-SPY1 dataset: (x × y × z—0.7 mm, 0.7 mm, 1.5 mm); NSCLC IO dataset: (x × y × z—0.54 mm, 0.54 mm, 0.8 mm)]. The variation in the contrast enhancement and kernel resolution parameters is addressed by harmonizing the features from the above resampled images, using the image acquisition parameters as batch variables in this scenario.
4. The variation in image physical dimensions and acquisition parameters is addressed by harmonizing the radiomic features using the voxel spacing parameters, contrast enhancement and kernel resolution parameters as the batch variables. This is performed under two scenarios, using offsets of 3 mm (4A) or 5 mm (4B) while extracting the features.

**Table 3:** A description of the heterogeneity-mitigation scenarios.

Scenario	Description
Original	No mitigation of heterogeneity performed on original radiomic features
1A	Offset 3 mm for feature extraction, harmonize by voxel spacing parameters
1B	Offset 5 mm for feature extraction, harmonize by voxel spacing parameters
2A	Offset 3 mm for feature extraction, harmonize by image acquisition parameters
2B	Offset 5 mm for feature extraction, harmonize by image acquisition parameters
3	Resample to minimum voxel spacing and harmonize by image acquisition parameters
4A	Offset 3 mm for feature extraction, harmonize by voxel spacing and image acquisition parameters
4B	Offset 5 mm for feature extraction, harmonize by voxel spacing and image acquisition parameters

#### Radiomic phenotype identification

Following heterogeneity-mitigation with each of the scenarios described above, unsupervised hierarchical clustering was performed on the features^32^. An agglomerative approach was used to create a hierarchical clustering of the patients using Euclidean distance between the extracted features and Ward’s minimum variance method as the clustering criterion^33^. The optimal number of distinct phenotypes, k, was determined by assessing the stability and significance of each phenotype for each value of k that was considered. The optimal number of stable phenotypes was determined using consensus clustering^34^, where dataset was sub-sampled and cluster arrangements were determined using varying values of k. For each value of k, the proportion that two patients occupied the same phenotype cluster out of the number of times they appeared in the same subsample was determined and stored in a consensus matrix, from which a cumulative distribution function (CDF) was determined. Cluster stability, determined by the area under the CDF curve, was evaluated for each value of k. Statistical significance of the identified, stable phenotypes was evaluated using the SigClust method^35,36^. Here, the significance of the cluster index, defined as the sum of within-cluster sums of squares about the cluster-mean divided by the total sum of squares about the overall mean was tested against a null distribution, simulated using 10,000 samples from a Gaussian distribution fit to the data. The test was performed at each phenotype split to determine statistical significance (p < 0.05). Two optimal radiomic phenotypes were identified in each scenario.

#### Details of prognostic models and their association with survival outcome

**Figure 1:**
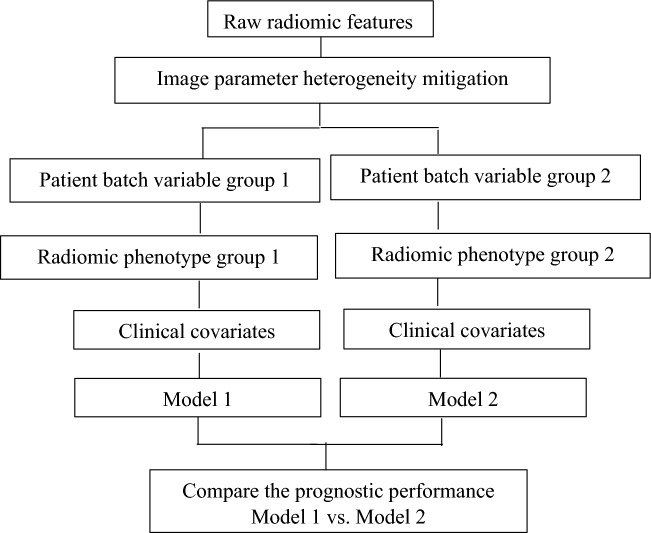
Steps involved in comparing the prognostic scores of the models derived from the patient groups divided based on individual batch variables, to assess the reproducibility of the prognostic scores.

The patients were divided into groups based on individual batch variables. For each group, the phenotype derived from the radiomic features is combined with the clinical covariates into a prognostic model. The clinical covariates for the breast ISPY dataset (Supplementary Table S2) include age, HR Pos (Hormone Receptor status) and HER2 Most Pos (Her2 status) and the clinical covariates for the lung IO dataset (Supplementary Table S4) include PDL1 expression, ECOG, BMI and smoking status. The prognostic performance of the groups is assessed using the concordance statistic (c-statistic)^37^, derived from a Cox proportional hazards model fitted on the all the patients present within a given group (Table 4: Breast I-SPY1 and Table 5: NSCLC IO). We also wanted to see if the trends in the c-statistics hold when c-scores are derived from a five-fold cross-validated Cox proportional-hazards analysis with 200 iterations. These cross-validated c-scores and 95% confidence intervals (CIs) have been included in the Supplementary File (Table S7: breast I-SPY1 and Table S8: NSCLC IO). A flowchart summarizing the steps involved in comparing the prognostic performance of the models derived from the patient groups is included below (Fig. 1).

**Table 4:** Prognostic performance of patient groups divided on the basis of batch variables for models derived from raw and heterogeneity-mitigated features: Breast I-SPY1 dataset.

Patient group	Number of patients	Original c-score	1A c-score	1B c-score	2A c-score	2B c-score	3 c-score	4A c-score	4B c-score
**Breast I-SPY1**									
Small pixel spacing	57	0.54	0.58	0.57	0.56	0.55	0.58	0.56	0.57
Large pixel spacing	99	0.65	0.64	0.63	0.65	0.64	0.67	0.63	0.64
Thin slice	74	0.67	0.67	0.61	0.63	0.62	0.62	0.62	0.63
Thick slice	82	0.52	0.52	0.54	0.56	0.55	0.55	0.54	0.55

**Table 5:** Prognostic performance of patient groups divided on the basis of batch variables for models derived from raw and heterogeneity-mitigated features: NSCLC IO dataset.

Patient group	Number of patients	Original c-score	1A c-score	1B c-score	2A c-score	2B c-score	3 c-score	4A c-score	4B c-score
**NSCLC IO**									
Contrast enhanced	80	0.63	0.65	0.64	0.64	0.63	0.65	0.64	0.65
Non-contrast enhanced	27	0.55	0.52	0.51	0.53	0.54	0.53	0.51	0.54
Small pixel spacing	58	0.67	0.62	0.63	0.61	0.64	0.64	0.63	0.62
Large pixel spacing	49	0.61	0.60	0.60	0.59	0.60	0.60	0.62	0.60
Low kernel resolution	17	0.57	0.59	0.59	0.59	0.59	0.61	0.60	0.69
High kernel resolution	90	0.62	0.63	0.65	0.65	0.63	0.66	0.67	0.65
Thin slice	64	0.66	0.61	0.62	0.62	0.62	0.62	0.59	0.63
Thick slice	43	0.56	0.58	0.58	0.58	0.59	0.60	0.57	0.60

The comparison is performed for the models containing the radiomic biomarkers derived from the features where the image parameter heterogeneity has been mitigated using various scenarios and the model containing the radiomic biomarker derived from the raw features.

## Results

In our analysis, we have compared the performance of the prognostic models in the patient groups divided on the basis of the individual batch variables. The mean prognostic score (central tendency) of the first group’s model is compared to the second group’s model.

We made the following observations:

1. For the I-SPY1 and IO datasets, scenario 3 (radiomic features, derived from images that have been resampled to the minimum voxel spacing, are harmonized using the image acquisition parameters as batch variables) gave models with consistently high prognostic scores across the batch variable groups (I-SPY1 dataset (batch variable group: large pixel spacing)—c-score: 0.67, IO dataset (batch variable group: high kernel resolution)—c-score: 0.66).
2. The prognostic performance of the patient groups divided on the basis of batch variables is not comparable in the case of models built using the raw, non-heterogeneity mitigated features (for instance: I-SPY1 dataset (batch variable group: small pixel spacing)—c-score: 0.54, (batch variable group: large pixel spacing)—c-score: 0.65, IO dataset (batch variable group: low kernel resolution)—c-score: 0.57, (batch variable group: high kernel resolution)—c-score: 0.62).
3. The prognostic performance of the patient groups divided on the batch variables are closer (comparable) in case of pixel spacing for the I-SPY1 dataset (for instance scenario 1A—batch variable group: small pixel spacing—c-score: 0.58, large pixel spacing—c-score: 0.64) and slice thickness for the IO dataset (for instance scenario 3—batch variable group: thin slice—c-score: 0.62, thick slice—c-score: 0.60).
  The prognostic performance of the models fitted on the entire dataset, for both the raw and heterogeneity-mitigated features, has been included in Supplementary Table S11 (for the ISPY1 dataset) and Table S12 (for the IO dataset).
4. The p value of the dendrogram split is more significant in the heatmaps derived from the heterogeneity mitigated features as compared to the non-heterogeneity mitigated features (for instance, in Table 6 (Breast I-SPY1 dataset), the p value of the dendrogram split in the heatmap for the non-heterogeneity mitigated features (patient group: thick slices) is 0.04 and 0.001 in the heatmap for features with heterogeneity mitigated using scenario 4b (harmonize by voxel spacing and image acquisition parameters, offset 5 mm for feature extraction).
  The p value of the dendrogram splits for the patient groups based on the other batch variables (for the Breast I-SPY1 and NSCLC IO datasets) are included in the Supplementary Table S9.
5. The normalized mutual information (NMI) between phenotypes derived from heterogeneity mitigation scenario 3 (models with the highest prognostic scores) and other heterogeneity mitigation scenarios is higher than the NMI between phenotypes derived from heterogeneity mitigation scenario 3 and those derived from the non-heterogeneity mitigated scenario (for instance, in Table 7 (NSCLC IO dataset), for patients with high kernel resolution scans, the NMI between phenotypes derived from scenario 3 and scenario 1a (harmonize by voxel spacing parameters, offset 3 mm for feature extraction) is 0.38 and the NMI between scenario 3 and those derived from the non-heterogeneity mitigated scenario is 0.003).

**Table 6:** Significance of the cluster dendrogram split for heatmaps built using features subjected to various heterogeneity mitigation and non-mitigation scenarios; for patient groups divided based on batch variables (thick slice: Breast I-SPY1 dataset; high kernel resolution: NSCLC IO dataset).

Thick slice: Breast I-SPY1		High kernel resolution: NSCLC IO	
Scenario	p value dendrogram	Scenario	p value dendrogram
1a	0.004	1a	0.001
1b	0.003	1b	0.02
2a	0.01	2a	0.015
2b	0.02	2b	0.003
3	0.005	3	0.0004
4a	0.03	4a	0.0011
4b	0.001	4b	0.0007
Non-mitigated	0.04	Non-mitigated	0.02

**Table 7:** Normalized mutual information between phenotypes of the best-performing heterogeneity-mitigation scenario and other mitigation and non-mitigation scenarios; for patient groups divided based on batch variables (thick slice: Breast I-SPY1 dataset; high kernel resolution: NSCLC IO dataset).

Thick slice: Breast I-SPY1		High kernel resolution: NSCLC IO	
Scenario	p value dendrogram	Scenario	p value dendrogram
3 vs. non-mitigated	0.002	3 vs. non-mitigated	0.003
3 vs. 1a	0.23	3 vs. 1a	0.38
3 vs. 1b	0.17	3 vs. 1b	0.11
3 vs. 2a	0.21	3 vs. 2a	0.25
3 vs. 2b	0.17	3 vs. 2b	0.37
3 vs. 4a	0.18	3 vs. 4a	0.28
3 vs. 4b	0.22	3 vs. 4b	0.30

The NMI values between phenotypes for the patient groups based on the other batch variables (for the Breast I-SPY1 and NSCLC IO datasets) are included in the Supplementary Table S10.

**Figure 2:**
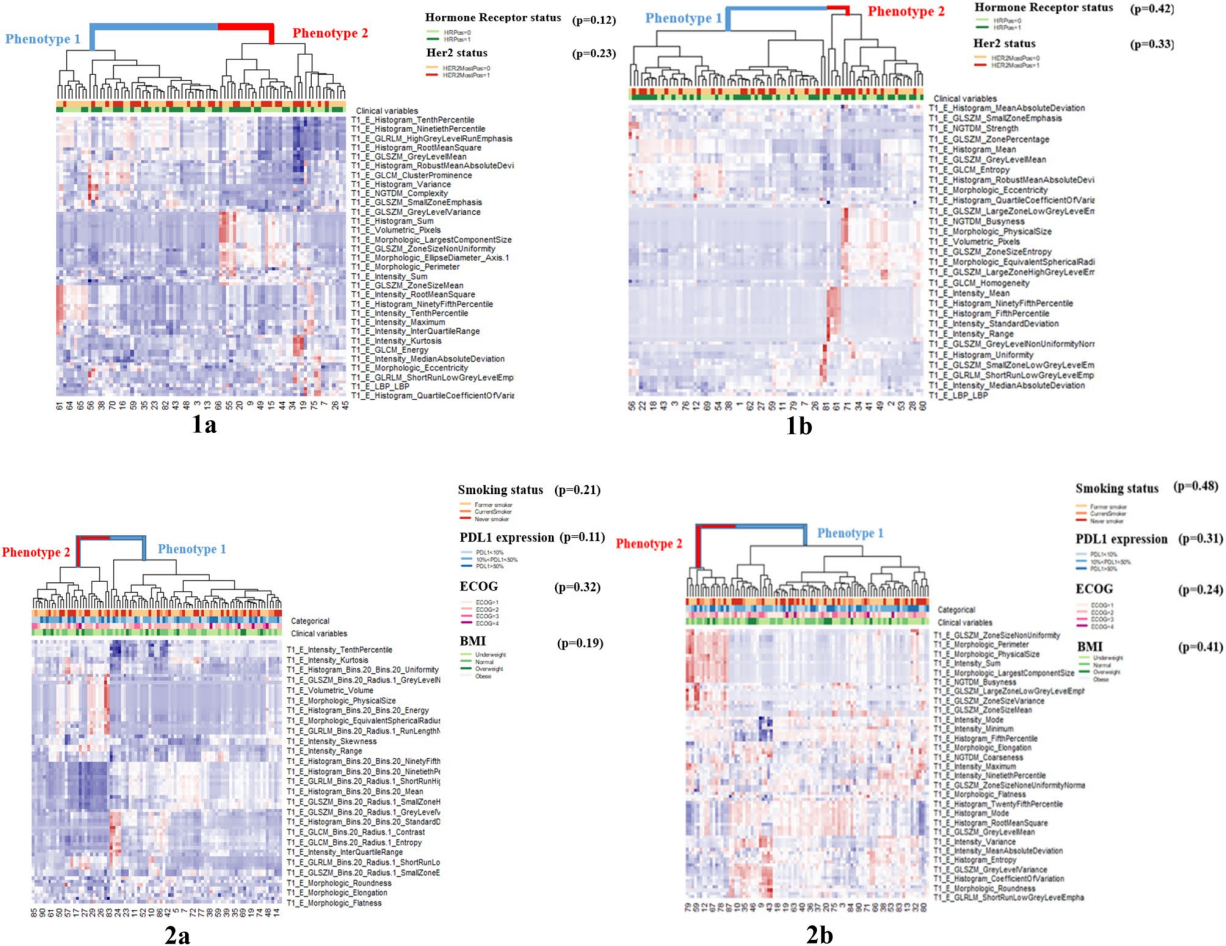
Heatmap of radiomic derived features (created using R programing language (ver. 3.5.1) https://www.r-project.org/). Unsupervised hierarchical clustering in the patients grouped based on batch variables for heterogeneity mitigated features (scenario 3: radiomic features, derived from images that have been resampled to the minimum voxel spacing, are harmonized using the image acquisition parameters as batch variables) and non-heterogeneity mitigated features identifies two distinct and statistically significant tumor radiomic phenotypes for breast I-SPY1 dataset (patients with thick slices: (**1a**) (heterogeneity mitigated features heatmap), p value-0.005; and (**1b**) (non-heterogeneity mitigated features heatmap), p value-0.04; and NSCLC IO dataset (patients with high kernel resolution images: (**2a**) (heterogeneity mitigated features heatmap), p value-0.0004; and (**2b**) (non-heterogeneity mitigated features heatmap), p value-0.02. Association of these phenotypes with the clinical covariates is assessed by the Chi square test and the resultant p values are included in the figure.

The phenotypes from the radiomic features mitigated using scenario 3 and the non-heterogeneity mitigated features for patients grouped based on their batch variables can be visualized using Fig. 2 [(panel 1a: Breast I-SPY1 dataset-patients with thick slices, heterogeneity mitigated features), (panel 1b: Breast I-SPY1 dataset—patient with thick slices, non-heterogeneity mitigated features), (panel 2a: NSCLC IO dataset—patients with high kernel resolution images, heterogeneity mitigated features), (panel 2b: NSCLC IO dataset—patients with high kernel resolution images, non-heterogeneity mitigated features)].

## Discussion

The heterogeneous nature of image parameters, as a result of variation in scanner parameters and image acquisition protocols, especially in large-scale retrospective datasets from multi-institutional studies, makes the development of reproducible radiomic biomarkers challenging. The radiomics community has recently started discussing how the robustness of radiomic biomarkers to the heterogeneity in image parameters is essential to improving their acceptance in the clinical community. However, even though previous studies have focused on the reproducibility of radiomic features in a limited setting (test–retest, phantom studies etc.) and have explored the sensitivity of individual radiomic features to image parameter variability, little attention has been given to assessing how this variability affects the reproducibility of radiomic signatures.

Our study assesses several techniques to address the effect of heterogeneity in image parameters on the reproducibility of radiomic biomarkers. We observed that, in case of both the databases, the phenotypes derived from features whose heterogeneity has been mitigated using various scenarios are more similar to each other (higher normalized mutual information (NMI) score). The NMI score is lower between phenotypes derived from heterogeneity-mitigated features and phenotypes derived from the raw features. In the non-heterogeneity mitigated models, the prognostic scores are not comparable across the patient groups divided on the basis of each batch variable. The prognostic performance of the patient groups divided based on the batch variables are closer (comparable) in case of pixel spacing for the Breast I-SPY1 dataset and voxel spacing for the NSCLC IO dataset. We note that, among the various heterogeneity mitigation scenarios, the model containing the radiomic phenotypes derived from scenario 3 (resampling images to minimum voxel spacing and harmonizing for differences in image acquisition parameters) had a higher prognostic performance across most of the patient groups, and thus can be used as a potential starting point for the heterogeneity mitigation component in future studies. Our results also show that the phenotypes obtained using unsupervised hierarchical clustering are more significant (metric—p value of dendrogram split in the heatmap) in the case of heterogeneity mitigated features compared to the raw features.

We note that although the statistical significance of the phenotypes obtained with heterogeneity mitigation is improved, the prognostic performance of the models does not improve substantially. One of the possible reasons for this reduction has been discussed in the paper based on the harmonization method used in our analysis: “A possible explanation is that because imaging parameters were generally associated with outcome as a consequence is study design, the removal of variation associated with those imaging parameters reduced predictive performance”^30^. However, we would like to point out that an improvement in the reproducibility of the radiomic signatures does not necessarily correlate with an improvement in prognostic performance. Although the predictive performance may be moderate for some of our radiomic models, the application of heterogeneity-mitigation techniques does make the prognostic scores more comparable across the patient groups divided based on the batch variables, and hence, reproducible. We would like to reinstate here that it is more desirable to have a model with a modest prognostic performance in the training set, but with comparable performance in the test set, as compared to having a model with high performance only in the training set, but the performance does not validate in the test set. Even a radiomic biomarker with a high prognostic performance loses interpretability if it is highly sensitive to changes in image parameters. Reproducibility of the biomarker is key to make it usable in a clinical setting.

Our results indicate that accounting for heterogeneity in image parameters is important to obtain more reproducible prognostic scores, irrespective of the image site or modality. Our study discusses the importance of heterogeneity mitigation in radiomic parameters and why it is important to ensure that the prognostic model is robust to the variation in image acquisition and physical dimensions. It also addresses the question of post-processing feature standardization, as standardization during the image acquisition stage might not be feasible, especially in large datasets obtained from multi-institutional studies. We hope our study can be a step in the direction of constructing reproducible radiomic biomarkers, thus increasing their application and acceptance in clinical decision-making.

## Supplementary Information

Supplementary Information.

## Data Availability

The datasets used and/or analyzed during the current study are available from the corresponding author on reasonable request.

## Abbreviations

DCE: Dynamic contrast enhanced
HR: Hormone receptor status
IO: Immunotherapy
NSCLC: Non-small cell lung cancer
PDL1: Programmed death-ligand 1
PEMBRO: Pembrolizumab
NSCLC: Non-small cell lung cancer
RFS: Recurrence-free survival
ROI: Region of interest
ECOG: Eastern Cooperative Oncology Group
BMI: Body mass index

## References

[1] La Thangue NB, Kerr DJ (2011). Predictive biomarkers: A paradigm shift towards personalized cancer medicine. Nat. Rev. Clin. Oncol..

[2] Ding L, Wendl MC, Koboldt DC, Mardis ER (2010). Analysis of next-generation genomic data in cancer: Accomplishments and challenges. Hum. Mol. Genet..

[3] Vaidyanathan R, Soon RH, Zhang P, Jiang K, Lim CT (2019). Cancer diagnosis: From tumor to liquid biopsy and beyond. Lab on a Chip.

[4] Kuo MD, Jamshidi N (2014). Behind the numbers: Decoding molecular phenotypes with radiogenomics—Guiding principles and technical considerations. Radiology.

[5] O’Connor JP, Aboagye EO, Adams JE, Aerts HJ, Barrington SF, Beer AJ (2017). Imaging biomarker roadmap for cancer studies. Nat. Rev. Clin. Oncol..

[6] Lambin P, Leijenaar RT, Deist TM, Peerlings J, De Jong EE, Van Timmeren J, Sanduleanu S, Larue RT, Even AJ, Jochems A, van Wijk Y (2017). Radiomics: The bridge between medical imaging and personalized medicine. Nat. Rev. Clin. Oncol..

[7] Lambin P, Rios-Velazquez E, Leijenaar R, Carvalho S, Van Stiphout RG, Granton P (2012). Radiomics: Extracting more information from medical images using advanced feature analysis. Eur. J. Cancer.

[8] Zhao B (2021). Understanding sources of variation to improve the reproducibility of radiomics. Front. Oncol..

[9] Huang YQ, Liang CH, He L, Tian J, Liang CS, Chen X (2016). Development and validation of a radiomics nomogram for preoperative prediction of lymph node metastasis in colorectal cancer. J. Clin. Oncol..

[10] Velazquez ER, Aerts HJ, Oberije C, De Ruysscher D, Lambin P (2010). Prediction of residual metabolic activity after treatment in NSCLC patients. Acta Oncol..

[11] Strimbu K, Tavel JA (2010). What are biomarkers?. Curr. Opin. HIV AIDS.

[12] Davis AT, Palmer AL, Pani S, Nisbet A (2018). Assessment of the variation in CT scanner performance (image quality and Hounsfield units) with scan parameters, for image optimisation in radiotherapy treatment planning. Phys. Med..

[13] Traverso A, Wee L, Dekker A, Gillies R (2018). Repeatability and reproducibility of radiomic features: A systematic review. Int. J. Radiat. Oncol. Biol. Phys..

[14] Aerts HJ, Velazquez ER, Leijenaar RT, Parmar C, Grossmann P, Carvalho S (2014). Decoding tumour phenotype by noninvasive imaging using a quantitative radiomics approach. Nat. Commun..

[15] Armato SG, Meyer CR, McNitt-Gray MF, McLennan G, Reeves AP, Croft BY (2008). The reference image database to evaluate response to therapy in lung cancer (RIDER) project: A resource for the development of change-analysis software. Clin. Pharmacol. Therap..

[16] van Timmeren JE, Leijenaar RT, van Elmpt W, Wang J, Zhang Z, Dekker A, Lambin P (2016). Test–retest data for radiomics feature stability analysis: Generalizable or study-specific?. Tomography.

[17] Zhovannik I, Bussink J, Traverso A, Shi Z, Kalendralis P, Wee L (2019). Learning from scanners: Bias reduction and feature correction in radiomics. Clin. Transl. Radiat. Oncol..

[18] Midya A, Chakraborty J, Gönen M, Do RK, Simpson AL (2018). Influence of CT acquisition and reconstruction parameters on radiomic feature reproducibility. J. Med. Imaging.

[19] Mackin D, Fave X, Zhang L, Fried D, Yang J, Taylor B (2015). Measuring CT scanner variability of radiomics features. Investig. Radiol..

[20] Johnson WE, Li C, Rabinovic A (2007). Adjusting batch effects in microarray expression data using empirical Bayes methods. Biostatistics.

[21] Ibrahim A, Refaee T, Leijenaar RT, Primakov S, Hustinx R, Mottaghy FM (2021). The application of a workflow integrating the variable reproducibility and harmonizability of radiomic features on a phantom dataset. PLoS ONE.

[22] Newitt D, Hylton N (2016). Multi-center breast DCE-MRI data and segmentations from patients in the I-SPY 1/ACRIN 6657 trials. Cancer Imaging Arch..

[23] Clark K, Vendt B, Smith K, Freymann J, Kirby J, Koppel P (2013). The cancer imaging archive (TCIA): Maintaining and operating a public information repository. J. Dig. Imaging.

[24] Singh A, Horng H, Roshkovan L, Weeks JK, Hershman M, Noël P (2022). Development of a robust radiomic biomarker of progression-free survival in advanced non-small cell lung cancer patients treated with first-line immunotherapy. Sci. Rep..

[25] Yushkevich, P. A., Gao, Y. & Gerig, G. ITK-SNAP: An interactive tool for semi-automatic segmentation of multi-modality biomedical images. In *2016 38th Annual International Conference of the IEEE Engineering in Medicine and Biology Society (EMBC)*, 3342–3345 (IEEE, 2016). 10.1109/EMBC.2016.7591443 PMC5493443 28269019

[26] Rathore, S., Bakas, S., Pati, S., Akbari, H., Kalarot, R., Sridharan, P. *et al*. Brain cancer imaging phenomics toolkit (brain-CaPTk): An interactive platform for quantitative analysis of glioblastoma. In *International MICCAI Brainlesion Workshop*, 133–145 (Springer, 2017). 10.1007/978-3-319-75238-9_12 PMC5934754 29733087

[27] Zwanenburg A, Vallières M, Abdalah MA, Aerts HJ, Andrearczyk V, Apte A (2020). The image biomarker standardization initiative: Standardized quantitative radiomics for high-throughput image-based phenotyping. Radiology.

[28] Fortin JP, Cullen N, Sheline YI, Taylor WD, Aselcioglu I, Cook PA (2018). Harmonization of cortical thickness measurements across scanners and sites. Neuroimage.

[29] Horng H, Singh A, Yousefi B, Cohen EA, Haghighi B, Katz S (2022). Generalized ComBat harmonization methods for radiomic features with multi-modal distributions and multiple batch effects. Sci. Rep..

[30] Horng H, Singh A, Yousefi B, Cohen EA, Haghighi B, Katz S (2022). Improved generalized ComBat methods for harmonization of radiomic features. Sci. Rep..

[31] https://github.com/hannah-horng/opnested-combat. Accessed May 1 2022.

[32] Kassambara A (2017). Practical Guide to Cluster Analysis in R: Unsupervised Machine Learning.

[33] Ward JH (1963). Hierarchical grouping to optimize an objective function. J. Am. Stat. Assoc..

[34] Monti S, Tamayo P, Mesirov J, Golub T (2003). Consensus clustering: A resampling-based method for class discovery and visualization of gene expression microarray data. Mach. Learn..

[35] Liu Y, Hayes DN, Nobel A, Marron JS (2008). Statistical significance of clustering for high-dimension, low-sample size data. J. Am. Stat. Assoc..

[36] Chitalia RD, Rowland J, McDonald ES, Pantalone L, Cohen EA, Gastounioti A (2020). Imaging phenotypes of breast cancer heterogeneity in preoperative breast dynamic contrast enhanced magnetic resonance imaging (DCE-MRI) scans predict 10-year recurrence radiomic phenotypes of tumor heterogeneity. Clin. Cancer Res..

[37] Uno H, Cai T, Pencina MJ, D’Agostino RB, Wei LJ (2011). On the C-statistics for evaluating overall adequacy of risk prediction procedures with censored survival data. Stat. Med..

