# Supplementary material for: Resampling and harmonization for mitigation of heterogeneity in image parameters of baseline scans
Source: Sci Rep. 2022 Dec 13;12:21505. doi: 10.1038/s41598-022-26083-4 (PMC9747915; doi:10.1038/s41598-022-26083-4)
Supplement: Supplementary file 1 — Supplementary Information. [file 41598_2022_26083_MOESM1_ESM.docx]

**Resampling and harmonization for mitigation of heterogeneity in image parameters of baseline scans**

Apurva Singh^1,2^, Hannah Horng^2^, Rhea Chitalia^1,2^, Leonid Roshkovan^1^, Sharyn I. Katz^1^, Peter Noël^1^, Russell T. Shinohara^3^, Despina Kontos^1^

^1^Department of Radiology, University of Pennsylvania, Philadelphia, PA, 19104

^2^Department of Bioengineering, University of Pennsylvania, Philadelphia, PA, 19104

^3^Department of Biostatistics, Epidemiology, and Informatics, Philadelphia, PA, 19104

**
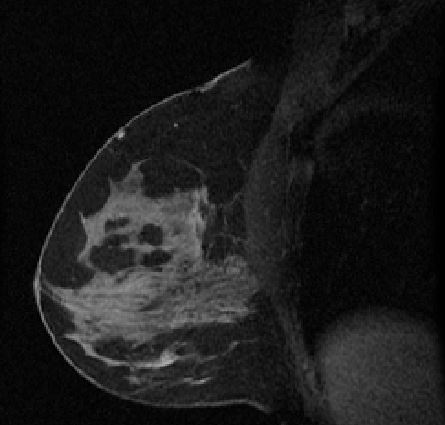

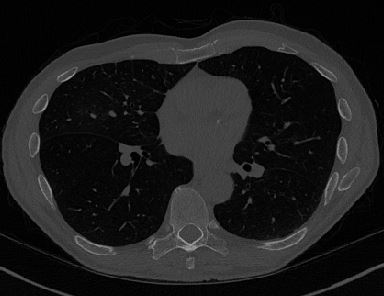
**

**Figure S1: Representative images from I-SPY1 (DCE-MRI) and NSCLC IO (CT) datasets.**

**Table S1: Patient demographic information: Breast I-SPY1 dataset**

| **Demographic variable** | **Category** | **Number of patients**  **(n=156)** |
| --- | --- | --- |
| **Race** | Unknown | 2 (1.3%) |
|  | Caucasian | 118 (75.6%) |
|  | African American | 27 (17.4%) |
|  | Asian | 7 (4.5%) |
|  | Native Hawaiian/Pacific Islander | 1 (0.6%) |
|  | Multiple race | 1 (0.6%) |
| **Age (years)** | Median, Range | 49 [28,69] |

**Table S2: Clinical covariate categories and number of patients: Breast I-SPY1 dataset**

| **Clinical covariate** | **Category** | **Number of patients**  **(n=156)** |
| --- | --- | --- |
| **HR Pos** | Hormone Receptor Status pre-treatment:  Negative for both ER and PR  Hormone Receptor Status pre-treatment:  Positive if either ER or PR was positive | 67 (42.9%)  89 (57.1%) |
| **HER2MostPos** | HER2 Status pre-treatment: Negative  HER2 Status pre-treatment: Positive | 106 (67.9%)  50 (32.1%) |

**Table S3: Patient demographic information: NSCLC IO dataset**

| **Demographic variable** | **Category** | **Number of patients**  **(n=107)** |
| --- | --- | --- |
| **Sex** | Male  Female | 52 (48.6%)  55 (51.4%) |
| **Race** | White  Black or African American  Latino  Asian  Other | 73 (68.2%)  29 (27.1%)  1 (0.9%)  1 (0.9%)  3 (2.8%) |
| **Age (years)** | Median, Range | 67, [38,90] |

**Table S4: Clinical covariate categories and number of patients: NSCLC IO dataset**

| **Clinical covariate** | **Category** | **Number of patients**  **(n=107)** |
| --- | --- | --- |
| **PDL1 expression** | PDL1 < 10%  10%≤ PDL1<50%  PDL1≥50% | 60 (56.1%)  18 (16.8%)  29 (27.1%) |
| **BMI** | Underweight (BMI<18.5)  Normal (18.5≤BMI≤24.9)  Overweight (25≤BMI≤29.9)  Obese (BMI≥30) | 2 (1.8%)  37 (34.6%)  39 (36.4%)  29 (27.1%) |
| **Smoking Status** | Former smoker  Current smoker  Non-smoker | 54 (50.5%)  39 (36.4%)  14 (13.1%) |
| **ECOG Performance Status** | Value 1  Value 2  Value 3  Value 4 | 35 (32.7%)  51 (47.7%)  14 (13.1%)  7 (6.5%) |

**Table S5: Percentage of features with significantly different distributions attributable to batch effects in the original features and after heterogeneity-mitigation using various scenarios, detected with AD test at a significance level of p < 0.05, for the breast I-SPY1 dataset.**

**1A- Harmonize by voxel spacing parameters, offset 3mm for feature extraction.**

| **Batch variable** | **Original features** | **Heterogeneity-mitigated features** |
| --- | --- | --- |
| Pixel spacing | 50% | 44.8% |
| Slice thickness | 20% | 11.4% |

**1B- Harmonize by voxel spacing parameters, offset 5mm for feature extraction.**

| **Batch variable** | **Original features** | **Heterogeneity-mitigated features** |
| --- | --- | --- |
| Pixel spacing | 50% | 45% |
| Slice thickness | 20% | 10.5% |

**2A- Harmonize by image acquisition parameters, offset 3mm for feature extraction.**

| **Batch variable** | **Original features** | **Heterogeneity-mitigated features** |
| --- | --- | --- |
| Site | 97.1% | 8% |
| Manufacturer | 66.2% | 5.2% |
| Model name | 82.4% | 3.8% |

**2B- Harmonize by image acquisition parameters, offset 5mm for feature extraction.**

| **Batch variable** | **Original features** | **Heterogeneity-mitigated features** |
| --- | --- | --- |
| Site | 97.1% | 9% |
| Manufacturer | 66.2% | 5% |
| Model name | 82.4% | 3.3% |

**3- Resample to minimum voxel spacing and harmonize by image acquisition parameters.**

| **Batch variable** | **Original features** | **Heterogeneity-mitigated features** |
| --- | --- | --- |
| Site | 97.1% | 7.6% |
| Manufacturer | 66.2% | 5.7% |
| Model name | 82.4% | 9.5% |

**4A- Harmonize by voxel spacing and image acquisition parameters, offset 3mm for feature extraction.**

| **Batch variable** | **Original features** | **Heterogeneity-mitigated features** |
| --- | --- | --- |
| Pixel spacing | 50% | 0.9% |
| Slice thickness | 20% | 0.5% |
| Site | 97.1% | 4.3% |
| Manufacturer | 66.2% | 3.8% |
| Model name | 82.4% | 3.3% |

**4B- Harmonize by voxel spacing and image acquisition parameters, offset 5mm for feature extraction.**

| **Batch variable** | **Original features** | **Heterogeneity-mitigated features** |
| --- | --- | --- |
| Pixel spacing | 50% | 0.9% |
| Slice thickness | 20% | 0.7% |
| Site | 97.1% | 3.3% |
| Manufacturer | 66.2% | 4.3% |
| Model name | 82.4% | 3.1% |

**Table S6: Percentage of features with significantly different distributions attributable to batch effects in the original features and after heterogeneity-mitigation using various scenarios, detected with AD test at a significance level of p < 0.05, for the NSCLC IO dataset.**

**1A- Harmonize by voxel spacing parameters, offset 3mm for feature extraction.**

| **Batch variable** | **Original features** | **Heterogeneity-mitigated features** |
| --- | --- | --- |
| Pixel spacing | 16.8% | 5% |
| Slice thickness | 22.7% | 5.9% |

**1B- Harmonize by voxel spacing parameters, offset 5mm for feature extraction.**

| **Batch variable** | **Original features** | **Heterogeneity-mitigated features** |
| --- | --- | --- |
| Pixel spacing | 16.8% | 3.7% |
| Slice thickness | 22.7% | 7.6% |

**2A- Harmonize by image acquisition parameters, offset 3mm for feature extraction.**

| **Batch variable** | **Original features** | **Heterogeneity-mitigated features** |
| --- | --- | --- |
| Contrast enhancement | 18.5% | 15.7% |
| Kernel resolution | 26.1% | 20.1% |

**2B- Harmonize by image acquisition parameters, offset 5mm for feature extraction.**

| **Batch variable** | **Original features** | **Heterogeneity-mitigated features** |
| --- | --- | --- |
| Contrast enhancement | 18.5% | 16.5% |
| Kernel resolution | 26.1% | 19.5% |

**3- Resample to minimum voxel spacing and harmonize by image acquisition parameters.**

| **Batch variable** | **Original features** | **Heterogeneity-mitigated features** |
| --- | --- | --- |
| Contrast enhancement | 18.5% | 13.6% |
| Kernel resolution | 26.1% | 19.6% |

**4A- Harmonize by voxel spacing and image acquisition parameters, offset 3mm for feature extraction.**

| **Batch variable** | **Original features** | **Heterogeneity-mitigated features** |
| --- | --- | --- |
| Pixel spacing | 16.8% | 5.9% |
| Slice thickness | 22.7% | 4.5% |
| Contrast enhancement | 18.5% | 14.3% |
| Kernel resolution | 26.1% | 18.5% |

**4B- Harmonize by voxel spacing and image acquisition parameters, offset 5mm for feature extraction.**

| **Batch variable** | **Original features** | **Heterogeneity-mitigated features** |
| --- | --- | --- |
| Pixel spacing | 16.8% | 4.7% |
| Slice thickness | 22.7% | 5.1% |
| Contrast enhancement | 18.5% | 11.8% |
| Kernel resolution | 26.1% | 20.2% |

Legend

| **Scenario** | **Description** |
| --- | --- |
| Original | No mitigation of heterogeneity performed on original radiomic features |
| 1A | Offset 3mm for feature extraction, harmonize by voxel spacing parameters |
| 1B | Offset 5mm for feature extraction, harmonize by voxel spacing parameters |
| 2A | Offset 3mm for feature extraction, harmonize by image acquisition parameters |
| 2B | Offset 5mm for feature extraction, harmonize by image acquisition parameters |
| 3 | Resample to minimum voxel spacing and harmonize by image acquisition parameters |
| 4A | Offset 3mm for feature extraction, harmonize by voxel spacing and image acquisition parameters |
| 4B | Offset 5mm for feature extraction, harmonize by voxel spacing and image acquisition parameters |

**Table S7: Prognostic performance of patient groups divided on the basis of batch variables for models derived from raw and heterogeneity-mitigated features: Breast I-SPY1 dataset.**

| **Patient group** | **Number of patients** | **Original**  **Five-fold cross-validated c-score, 95% CI** | **1A**  **Five-fold cross-validated c-score, 95% CI** | **1B**  **Five-fold cross-validated c-score, 95% CI** | **2A**  **Five-fold cross-validated c-score, 95% CI** | **2B**  **Five-fold cross-validated c-score, 95% CI** | **3**  **Five-fold cross-validated c-score, 95% CI** | **4A**  **Five-fold cross-validated c-score, 95% CI** | **4B**  **Five-fold cross-validated c-score, 95% CI** |
| --- | --- | --- | --- | --- | --- | --- | --- | --- | --- |
| Small pixel spacing | 57 | 0.49, [0.45,0.53] | 0.52, [0.51,0.58] | 0.53, [0.51,0.59] | 0.52, [0.51,0.59] | 0.51, [0.52,0.61] | 0.54, [0.52,0.61] | 0.50, [0.48,0.55] | 0.51, [0.50,0.57] |
| Large pixel spacing | 99 | 0.62, [0.50,0.64] | 0.60, [0.53,0.61] | 0.61, [0.53,0.62] | 0.60, [0.52,0.61] | 0.62, [0.54,0.64] | 0.62, [0.54,0.64] | 0.57, [0.50,0.58] | 0.58, [0.51,0.60] |
| Thin slice | 74 | 0.63, [0.50,0.66] | 0.57, [0.54,0.63] | 0.58, [0.52,0.63] | 0.58, [0.51,0.60] | 0.59, [0.53,0.63] | 0.59, [0.53,0.63] | 0.56, [0.51,0.60] | 0.58, [0.52,0.61] |
| Thick slice | 82 | 0.47, [0.44,0.52] | 0.49, [0.47,0.54] | 0.47, [0.46,0.54] | 0.49, [0.46,0.53] | 0.48, [0.50,0.61] | 0.48, [0.50,0.61] | 0.45, [0.44,0.51] | 0.47, [0.45,0.52] |

**Table S8: Prognostic performance of patient groups divided on the basis of batch variables for models derived from raw and heterogeneity-mitigated features: NSCLC IO dataset.**

| Patient group | Number of patients | Five-fold cross-validated c-score, 95% CI | Five-fold cross-validated c-score, 95% CI | Five-fold cross-validated c-score, 95% CI | Five-fold cross-validated c-score, 95% CI | Five-fold cross-validated c-score, 95% CI | Five-fold cross-validated c-score, 95% CI | Five-fold cross-validated c-score, 95% CI | Five-fold cross-validated c-score, 95% CI |
| --- | --- | --- | --- | --- | --- | --- | --- | --- | --- |
| Contrast enhanced | 80 | 0.59, [0.54,0.63] | 0.59, [0.53,0.63] | 0.59, [0.53,0.63] | 0.59, [0.53,0.63] | 0.58, [0.52,0.62] | 0.60, [0.55,0.64] | 0.58, [0.53,0.62] | 0.60, [0.56,0.63] |
| Non- contrast enhanced | 27 | 0.42, [0.39,0.54] | 0.46, [0.43,0.53] | 0.45, [0.43,0.52] | 0.46, [0.43,0.53] | 0.44, [0.43,0.53] | 0.46, [0.44,0.53] | 0.46, [0.43,0.57] | 0.48, [0.42,0.56] |
| Small pixel spacing | 58 | 0.62, [0.51,0.63] | 0.57, [0.52,0.61] | 0.58, [0.52,0.61] | 0.57, [0.52,0.61] | 0.59, [0.54,0.64] | 0.59, [0.53,0.62] | 0.58, [0.53,0.63] | 0.57, [0.53,0.62] |
| Large pixel spacing | 49 | 0.54, [0.45,0.61] | 0.53, [0.52,0.60] | 0.53, [0.50,0.62] | 0.54, [0.52,0.60] | 0.54, [0.50,0.60] | 0.54, [0.51,0.60] | 0.55, [0.52,0.61] | 0.54, [0.52,0.61] |
| Low kernel resolution | 17 | 0.43, [0.40,0.55] | 0.52, [0.50,0.58] | 0.53, [0.49,0.59] | 0.52, [0.50,0.59] | 0.51, [0.50,0.58] | 0.53, [0.50, 0.61] | 0.52, [0.50,0.59] | 0.53, [0.51,0.60] |
| High kernel resolution | 90 | 0.57, [0.50,0.65] | 0.59, [0.53,0.62] | 0.60, [0.53,0.63] | 0.59, [0.52,0.63] | 0.59, [0.52,0.60] | 0.61, [0.55,0.64] | 0.62, [0.55,0.63] | 0.60, [0.54,0.63] |
| Thin slice | 64 | 0.60, [0.54,0.63] | 0.56, [0.51,0.60] | 0.58, [0.52,0.63] | 0.55, [0.51,0.61] | 0.56, [0.50, 0.62] | 0.56, [0.53,0.62] | 0.54, [0.50,0.60] | 0.57, [0.52,0.62] |
| Thick slice | 43 | 0.52, [0.42,0.61] | 0.52, [0.50,0.59] | 0.53, [0.51,0.60] | 0.51, [0.50,0.59] | 0.52, [0.50, 0.61] | 0.54, [0.52,0.61] | 0.51, [0.50,0.59] | 0.53, [0.50,0.59] |

**Table S9: Significance of cluster dendrogram split for patient groups divided on basis of batch variables: Breast I-SPY1 and NSCLC IO datasets.**

**Thin slice- heterogeneity mitigation and non-mitigation scenarios: Breast I-SPY1**

**Large pixel- heterogeneity mitigation and non-mitigation scenarios: Breast I-SPY1**

| Scenario | P value dendrogram |
| --- | --- |
| 1a | 0.005 |
| 1b | 0.0008 |
| 2a | 0.003 |
| 2b | 0.0041 |
| 3 | 0.0008 |
| 4a | 0.0067 |
| 4b | 0.0005 |
| Non-mitigated | 0.01 |

| Scenario | P value dendrogram |
| --- | --- |
| 1a | 0.0006 |
| 1b | 0.001 |
| 2a | 0.0028 |
| 2b | 0.0002 |
| 3 | 0.003 |
| 4a | 0.0017 |
| 4b | 0.00003 |
| Non-mitigated | 0.049 |

**Small pixel- heterogeneity mitigation and non-mitigation scenarios: Breast I-SPY1**

| Scenario | P value dendrogram |
| --- | --- |
| 1a | 0.0042 |
| 1b | 0.006 |
| 2a | 0.004 |
| 2b | 0.002 |
| 3 | 0.0003 |
| 4a | 0.0007 |
| 4b | 0.0005 |
| Non-mitigated | 0.03 |

**Large pixel spacing- heterogeneity mitigation and non-mitigation scenarios: NSCLC IO**

**Low kernel resolution- heterogeneity mitigation and non-mitigation scenarios: NSCLC IO**

| Scenario | P value dendrogram |
| --- | --- |
| 1a | 0.001 |
| 1b | 0.02 |
| 2a | 0.015 |
| 2b | 0.003 |
| 3 | 0.0004 |
| 4a | 0.0011 |
| 4b | 0.0007 |
| Non- mitigated | 0.03 |

| Scenario | P value dendrogram |
| --- | --- |
| 1a | 0.039 |
| 1b | 0.03 |
| 2a | 0.015 |
| 2b | 0.01 |
| 3 | 0.02 |
| 4a | 0.003 |
| 4b | 0.0002 |
| Non-mitigated | 0.048 |

**Thick slice- heterogeneity mitigation and non-mitigation scenarios: NSCLC IO**

**Small pixel spacing- heterogeneity mitigation and non-mitigation scenarios: NSCLC IO**

| Scenario | P value dendrogram |
| --- | --- |
| 1a | 0.005 |
| 1b | 0.007 |
| 2a | 0.004 |
| 2b | 0.003 |
| 3 | 0.02 |
| 4a | 0.006 |
| 4b | 0.01 |
| Non-mitigated | 0.03 |

| Scenario | P value dendrogram |
| --- | --- |
| 1a | 0.001 |
| 1b | 0.02 |
| 2a | 0.015 |
| 2b | 0.003 |
| 3 | 0.0004 |
| 4a | 0.0011 |
| 4b | 0.0007 |
| Non-mitigated | 0.02 |

**Non-contrast enhancement- heterogeneity mitigation and non-mitigation scenarios: NSCLC IO**

**Thin slice- heterogeneity mitigation and non-mitigation scenarios: NSCLC IO**

| Scenario | P value dendrogram |
| --- | --- |
| 1a | 0.004 |
| 1b | 0.003 |
| 2a | 0.01 |
| 2b | 0.02 |
| 3 | 0.005 |
| 4a | 0.03 |
| 4b | 0.001 |
| Non-mitigated | 0.04 |

| Scenario | P value dendrogram |
| --- | --- |
| 1a | 0.001 |
| 1b | 0.02 |
| 2a | 0.015 |
| 2b | 0.003 |
| 3 | 0.0004 |
| 4a | 0.0011 |
| 4b | 0.0007 |
| Non-mitigated | 0.02 |

**Contrast enhancement- heterogeneity mitigation and non-mitigation scenarios: NSCLC IO**

| Scenario | P value dendrogram |
| --- | --- |
| 1a | 0.002 |
| 1b | 0.003 |
| 2a | 0.04 |
| 2b | 0.02 |
| 3 | 0.01 |
| 4a | 0.0002 |
| 4b | 0.004 |
| Non-mitigated | 0.03 |

**Table S10: Normalized mutual information between phenotypes of the best-performing heterogeneity-mitigation scenario and other mitigation and non-mitigation scenarios for patient groups divided on basis of batch variables: Breast I-SPY1 and NSCLC IO datasets.**

**Small pixel spacing: Breast I-SPY1**

**Large pixel spacing: Breast I-SPY1**

| **Scenario** | **Normalized Mutual information** |
| --- | --- |
| 3 vs. non-mitigated | 0.001 |
| 3 vs. 1a | 0.11 |
| 3 vs. 1b | 0.24 |
| 3 vs. 2a | 0.30 |
| 3 vs. 2b | 0.15 |
| 3 vs. 4a | 0.16 |
| 3 vs. 4b | 0.17 |

| **Scenario** | **Normalized Mutual information** |
| --- | --- |
| 3 vs. non-mitigated | 0.0005 |
| 3 vs. 1a | 0.12 |
| 3 vs. 1b | 0.14 |
| 3 vs. 2a | 0.11 |
| 3 vs. 2b | 0.15 |
| 3 vs. 4a | 0.13 |
| 3 vs. 4b | 0.17 |

**Thin slice: Breast I-SPY1**

| **Scenario** | **Normalized Mutual information** |
| --- | --- |
| 3 vs. non-mitigated | 0.003 |
| 3 vs. 1a | 0.12 |
| 3 vs. 1b | 0.28 |
| 3 vs. 2a | 0.19 |
| 3 vs. 2b | 0.11 |
| 3 vs. 4a | 0.23 |
| 3 vs. 4b | 0.18 |

**Low kernel resolution: NSCLC IO**

**Large pixel spacing: NSCLC IO**

| **Scenario** | **Normalized Mutual information** |
| --- | --- |
| 3 vs. non-mitigated | 0.02 |
| 3 vs. 1a | 0.19 |
| 3 vs. 1b | 0.11 |
| 3 vs. 2a | 0.15 |
| 3 vs. 2b | 0.41 |
| 3 vs. 4a | 0.29 |
| 3 vs. 4b | 0.15 |

| **Scenario** | **Normalized Mutual information** |
| --- | --- |
| 3 vs. non-mitigated | 0.029 |
| 3 vs. 1a | 0.13 |
| 3 vs. 1b | 0.58 |
| 3 vs. 2a | 0.13 |
| 3 vs. 2b | 0.73 |
| 3 vs. 4a | 0.21 |
| 3 vs. 4b | 0.14 |

**Thick slice: NSCLC IO**

**Small pixel spacing: NSCLC IO**

| **Scenario** | **Normalized Mutual information** |
| --- | --- |
| 3 vs. non-mitigated | 0.01 |
| 3 vs. 1a | 0.31 |
| 3 vs. 1b | 0.22 |
| 3 vs. 2a | 0.29 |
| 3 vs. 2b | 0.43 |
| 3 vs. 4a | 0.27 |
| 3 vs. 4b | 0.34 |

| **Scenario** | **Normalized Mutual information** |
| --- | --- |
| 3 vs. non-mitigated | 0.01 |
| 3 vs. 1a | 0.51 |
| 3 vs. 1b | 0.12 |
| 3 vs. 2a | 0.34 |
| 3 vs. 2b | 0.44 |
| 3 vs. 4a | 0.21 |
| 3 vs. 4b | 0.55 |

**Non-contrast enhanced: NSCLC IO**

**Thin slice: NSCLC IO**

| **Scenario** | **Normalized Mutual information** |
| --- | --- |
| 3 vs. non-mitigated | 0.02 |
| 3 vs. 1a | 0.19 |
| 3 vs. 1b | 0.25 |
| 3 vs. 2a | 0.15 |
| 3 vs. 2b | 0.24 |
| 3 vs. 4a | 0.31 |
| 3 vs. 4b | 0.14 |

| **Scenario** | **Normalized Mutual information** |
| --- | --- |
| 3 vs. non-mitigated | 0.02 |
| 3 vs. 1a | 0.59 |
| 3 vs. 1b | 0.27 |
| 3 vs. 2a | 0.45 |
| 3 vs. 2b | 0.60 |
| 3 vs. 4a | 0.62 |
| 3 vs. 4b | 0.45 |

**Contrast enhanced: NSCLC IO**

| **Scenario** | **Normalized Mutual information** |
| --- | --- |
| 3 vs. non-mitigated | 0.0003 |
| 3 vs. 1a | 0.21 |
| 3 vs. 1b | 0.13 |
| 3 vs. 2a | 0.16 |
| 3 vs. 2b | 0.36 |
| 3 vs. 4a | 0.18 |
| 3 vs. 4b | 0.19 |

**Table S11: Prognostic performance of the models fitted on the entire dataset, for both the raw and heterogeneity-mitigated features: Breast I-SPY1 dataset.**

| **Scenario** | **Description** | **c-score, 95% CI** | **Non cross-validated c-score** |
| --- | --- | --- | --- |
| Non-heterogeneity mitigated | No heterogeneity mitigation has been performed. | 0.54, [0.50,0.59] | 0.60 |
| 1A | Offset 3mm for feature extraction, harmonize by voxel spacing parameters. | 0.56, [0.51,0.61] | 0.62 |
| 1B | Offset 5mm for feature extraction, harmonize by voxel spacing parameters. | 0.57, [0.52,0.62] | 0.64 |
| 2A | Offset 3mm for feature extraction, harmonize by image acquisition parameters. | 0.54, [0.51,0.60] | 0.61 |
| 2B | Offset 5mm for feature extraction, harmonize by image acquisition parameters. | 0.53, [0.50,0.58] | 0.59 |
| 3 | Resample to minimum voxel spacing and harmonize by image acquisition parameters. | 0.59, [0.54,0.63] | 0.65 |
| 4A | Offset 3mm for feature extraction, harmonize by voxel spacing and image acquisition parameters. | 0.52, [0.45,0.59] | 0.58 |
| 4B | Offset 5mm for feature extraction, harmonize by voxel spacing and image acquisition parameters. | 0.53, [0.50,0.59] | 0.59 |

**Table S12: Prognostic performance of the models fitted on the entire dataset, for both the raw and heterogeneity-mitigated features: NSCLC IO dataset.**

| **Scenario** | **Description** | **C-score, 95% CI** | **Non cross-validated c-score** |
| --- | --- | --- | --- |
| Non-heterogeneity mitigated | No heterogeneity mitigation has been performed. | 0.58, [0.53,0.62] | 0.64 |
| 1A | Offset 3mm for feature extraction, harmonize by voxel spacing parameters. | 0.59, [0.52,0.60] | 0.65 |
| 1B | Offset 5mm for feature extraction, harmonize by voxel spacing parameters. | 0.61, [0.53,0.62] | 0.67 |
| 2A | Offset 3mm for feature extraction, harmonize by image acquisition parameters. | 0.58, [0.51,0.59] | 0.63 |
| 2B | Offset 5mm for feature extraction, harmonize by image acquisition parameters. | 0.57, [0.51,0.58] | 0.63 |
| 3 | Resample to minimum voxel spacing and harmonize by image acquisition parameters. | 0.63, [0.54,0.64] | 0.68 |
| 4A | Offset 3mm for feature extraction, harmonize by voxel spacing and image acquisition parameters. | 0.56, [0.50,0.57] | 0.61 |
| 4B | Offset 5mm for feature extraction, harmonize by voxel spacing and image acquisition parameters. | 0.60, [0.52,0.61] | 0.64 |

**Table S13**: A list of the radiomic features (102) extracted using CaPTk. The column headings indicate the family to which the list of features belongs to.

| **Intensity** | **Histogram** | **Volumetric** | **Morphologic** | **GLRLM** | **GLSZM** | **NGTDM** | **LBP** |
| --- | --- | --- | --- | --- | --- | --- | --- |
| Coefficient of Variation | Frequency | Pixels | Eccentricity | Grey Level Non- Uniformity Normalized | Grey Level Mean | Busyness | LBP |
| Energy | Coefficient of Variation | Volume | Ellipse Diameter | Grey Level Non-Uniformity | Grey Level Non- Uniformity | Coarseness |  |
| Inter Quartile Range | Energy |  | Elongation | Grey Level Variance | Grey Level Non-Uniformity Normalized | Complexity |  |
| Kurtosis | Entropy |  | Equivalent Spherical | High Grey Level Run Emphasis | Grey Level Variance | Contrast |  |
| Maximum | Fifth Percentile |  | Perimeter | Long Run Emphasis | High Grey Level Emphasis | Strength |  |
| Mean | Fifth Percentile Mean |  | Equivalent Spherical Radius | Long Run High Grey Level Emphasis | Large Zone Emphasis |  |  |
| Mean Absolute Deviation | Inter Quartile Range |  | Flatness | Long Run Low Grey Level Emphasis | Large Zone High Grey Level Emphasis |  |  |
| Median | Kurtosis |  | Largest Component Size | Low Grey Level Run Emphasis | Large Zone Low Grey Level Emphasis |  |  |
| Median Absolute Deviation | Mean |  | Number of Pixels | Run Entropy | Low Grey Level Emphasis |  |  |
| Minimum | Mean Absolute Deviation |  | Perimeter | Run Length Non- Uniformity Normalized | Small Zone Emphasis |  |  |
| Mode | Median |  | Physical Size | Run Length Non- Uniformity | Small Zone High Grey Level Emphasis |  |  |
| Ninetieth Percentile | Median Absolute Deviation |  | Roundness | Run Length Variance | Small Zone Low Grey Level Emphasis |  |  |
| Quartile Coefficient of Variation | Mode |  |  | Run Percentage | Zone Percentage |  |  |
| Range | Ninetieth Percentile |  |  | Short Run Emphasis | Zone Size Entropy |  |  |
| Root Mean Square | Ninety Fifth Percentile |  |  | Short Run High Grey Level Emphasis | Zone Size Mean |  |  |
| Skewness | Ninety Fifth Percentile Mean |  |  | Short Run Low Grey Level Emphasis | Zone Size Non- Uniformity |  |  |
| Standard Deviation | Quartile Coefficient of Variation |  |  | Total Runs | Zone Size Non-Uniformity Normalized |  |  |
| Sum | Robust Mean Absolute Deviation |  |  |  | Zone Size Variance |  |  |
| Tenth Percentile | Root Mean Square |  |  |  |  |  |  |
| Variance | Seventy Fifth Percentile |  |  |  |  |  |  |
|  | Skewness |  |  |  |  |  |  |
|  | Standard Deviation |  |  |  |  |  |  |
|  | Sum |  |  |  |  |  |  |
|  | Tenth Percentile |  |  |  |  |  |  |
|  | Twenty Fifth Percentile |  |  |  |  |  |  |
|  | Uniformity |  |  |  |  |  |  |
|  | Variance |  |  |  |  |  |  |

**Table S14**: Mathematical formulae describing the features.

| **Feature family** | **Description** |
| --- | --- |
| Intensity Features (First-Order Statistics) | - Minimum Intensity = Min (I_k_). where I_k_ is the intensity of pixel or voxel at index k. - Maximum Intensity = Max (I_k_). where I_k_ is the intensity of pixel or voxel at index k. - Mean= Σ(X_i_) where N is the number of   N  voxels/pixels.     - Standard Deviation = √(X-μ)^2^ where μ is the   N  mean of the data.   - Variance = (X-μ)^2^ where μ is the mean intensity.   N   - Skewness = Σ _i=1 to N (_X_i_-$\overline{X}$)^3^/N   s^3^  where $\overline{X}$ is the mean, s is the standard deviation and N is the number of pixels/voxels.   - Kurtosis = Σ _i=1 to N_ (X_i_-$\overline{X}$)^4^/N   s^4^  where  $\overline{X}$ is the mean, s is the standard deviation and N is the number of pixels/voxels. |
| Histogram-based features | - Uses number of bins as input and the number of pixels in each bin would be the output.   All features in this family are extracted from the discretized intensities. |
| Volumetric | - Volume/Area (depending on image dimension) and number of voxels/pixels in the ROI. |
| Morphologic | - Elongation = √(i_2_/i_1_) where in are the second moments of particle around its principal axes. - Perimeter = 2πr where r is the radius of the circle enclosing the shape. - Roundness = A_s_/A_c_= (Area of a shape)/ (Area of circle) where circle has the same perimeter. - Eccentricity = √[1- ((a+b)/c^2^)] where c is the longest semi-principal axis of an ellipsoid fitted on an ROI, and a and b are the 2nd and 3rd longest semi-principal axes of the ellipsoid. |
| GLRLM | For a given image, a run-length matrix P(i;j) is defined as the number of runs with pixels of gray level i and run length j.   - Short Run Emphasis (SRE) = 1 Σ_i,jtoN_ p(i,j)   n_r_ j^2^   - Long Run Emphasis (LRE) = 1 Σ_jtoN_ p(i,j).j^2^   n_r_   - Grey Level Non-uniformity (GLN) =   1 Σ_itoM_(Σ_jtoN_ p(i,j))^2^  n_r_   - Run Length Non-uniformity (RLN) =    1 Σ_i to M_ p_g_(i)  n_r_  i^2^     - High Grey-Level Run Emphasis   (HGRE)= 1 Σ_i to M_ p_g_(i). i^2^  n_r_   - Short Run Low Grey-Level Emphasis   (SRLGE)= 1 Σ_i to M_ Σ_j to N_ p(i,j)  n_r_ i^2^.j^2^   - Short Run High Grey-Level Emphasis   (SRHGE) = 1 Σ_i to M_ Σ_j to N_ p(i,j).i^2^  n_r_  j^2^   - Long Run Low Grey-Level Emphasis   (LRLGE) = 1 Σ_i to M_ Σ_j to N_ p(i,j).j^2^  n_r_  i^2^   - Long Run High Grey-Level Emphasis   (LRHGE) = 1 Σ_i to M_ Σ_j to N_ p(i,j).i^2^.j^2^  n_r_  All features are estimated within the ROI in an image, considering 26-connected neighbouring voxels in the 3D volume. |
| GLSZM | For a given image, a run-length matrix P(i;j) is defined as the number of runs with pixels of gray level i and run length j.   - Small Zone Emphasis (SZE) = 1 Σ_i,jtoN_ p(i,j)   n_r_ j^2^   - Large Zone Emphasis (LZE) = 1 Σ_jtoN_ p(i,j).j^2^   n_r_   - Gray-Level Non-uniformity (GLN) =   1 Σ_itoM_ (Σ_jtoN_ p(i,j))^2^  n_r_   - Zone-Size Non-uniformity (ZSN) =   1 Σ_jtoN_ (Σ_itoM_ p(i,j))^2^  n_r_   - Zone Percentage (ZP) = n_r_/n_p_ where n_r_ is the total number of runs and n_p_ is the number of pixels in the image. - Low Grey-Level Zone Emphasis (LGZE)=   1 Σ_i to M_ p_g_(i)  n_r_  i^2^   - High Grey-Level Zone Emphasis (HGZE)=   1 Σ_i to M_ p(i,j).i^2^  n_r_   - Short Zone Low Grey-Level Emphasis (SZLGE)=   1 Σ_i to M_ Σ_j to N_ p(i,j)  n_r_ i^2^. j^2^   - Short Zone High Grey-Level Emphasis (SZLGE) =   1 Σ_i to M_ Σ_j to N_ p(i,j).i^2^  n_r_  j^2^   - Long Zone Low Grey-Level Emphasis (LZLGE) =   1 Σ_i to M_ Σ_j to N_ p(i,j).j^2^  n_r_  i^2^   - Long Zone High Grey-Level Emphasis (LZHGE) =   1 Σ_i to M_ Σ_j to N_ p(i,j).i^2^.j^2^  n_r_ |
| NGTDM | - Coarseness = [ε +Σ _i=0toGk_ p_i_ s(i)] - Contrast =   [ 1 Σ_itoGk_ Σ_jtoGk_ pipj(i-j)^2^][1 Σ_itoGk_ s(i)]  Ns(Ns-1) n^2^   - Busyness =  [Σ_itoGk_ p_i_s(i)]   [Σ_itoGk_ Σ_itoGk_ ip_i_-jp_j_]   - Complexity = Σ_itoGk_ Σ_jtoGk_[(│i−j│)] [p_i_ s(i)+p_j_ s(j)]   (n^2^(p_i_+p_j_))   - Strength =    [Σ_itoGk_Σ_jtoGk_(p_i_+p_j_)(i-j)^2^]/[ε+Σ_itoGk_ s(i)]  Where pi is the probability of occurrence of a voxel of intensity i and s(i) represents the NGTDM value of intensity i calculated as: ∑│i−Ai│. Ai indicates the average intensity of the surrounding voxels without including the central voxel. |
| LBP | The pixel-wise LBP codes are computed using N number of neighbours on a circle of radius R around each pixel and using a rotation invariant implementation. The output value corresponds to the mean of the LBP map. |
